# Gambling Problems and Alexithymia: A Systematic Review

**DOI:** 10.3390/brainsci9080191

**Published:** 2019-08-07

**Authors:** Daniela Marchetti, Maria Cristina Verrocchio, Piero Porcelli

**Affiliations:** Department of Psychological, Health and Territorial Sciences, G. d’Annunzio University of Chieti-Pescara, 66100 Chieti, Italy

**Keywords:** gambling, alexithymia, review, community and clinical samples

## Abstract

Among the factors that are thought to underlie gambling problems, alexithymia has been recognized to contribute to their development. For the first time, we reviewed the literature on the relationship between alexithymia and gambling. A systematic search of literature was run in the major reference databases including PubMed, Cochrane Database for Systematic Review, PsycINFO, Web of Science, Scopus until April 2019. The search produced 182 articles that produced 20 papers included in the review. Fourteen studies were conducted with community samples of pathological gamblers while six studies with clinical samples of disordered gamblers. All studies assessed alexithymia with the Toronto Alexithymia Scale while gambling problems were assessed mostly with the South Oaks Gambling Screen. Alexithymic features were significantly more prevalent in pathological gamblers both at the community and clinical levels, increased symptom severity, and showed interactive mechanisms with personality, psychiatric, and cognitive factors. Alexithymia is likely to associate with gambling as a coping behavior to increase emotional arousal and avoid negative emotions, according to the affect dysregulation model. Further studies are needed to widen the knowledge on this association.

## 1. Introduction

After gambling opportunities have been spreading out around the world, gambling-related problems have received increased attention over the past three decades. Pathological gambling or gambling disorder and problem gambling are the two salient categories more often used in literature for identifying gambling-related problems. In the psychiatric classification, the diagnosis of pathological gambling was introduced in the Diagnostic and Statistical Manual of Mental Disorders, 3rd edition (DSM-III [1]) under the rubric of impulse control disorders and is now referred to as gambling disorder in the DSM-5 [2] under the rubric of addiction and substance use disorder. The two categories are used quite interchangeably in the literature. The term of problem gambling can be seen as a form of subclinical condition that is typically seen as a less severe form of gambling disorder [3].

Scientific understanding of this condition and its treatment has advanced extensively [4]. Gambling disorder is characterized by financial, psychological, employment, and relationship difficulties related to excessive wagering [2]. In severe cases, it can lead to legal problems and suicidal behaviors as well [5]. In a meta-analysis of prevalence studies, between 0.2% and 2.1% of the population develops gambling disorder and even a larger proportion (0.5–4.0%) experiences problem gambling [6].

Risk factors that may predispose individuals to problem gambling include demographic, environmental, personality and cognitive factors. Most identified risk factors include gender (men are exposed more than women [7]), socio-cultural background (environmental where gambling is accepted without stigma implications [8]), personality factors (impulsivity, sensation seeking, undercontrolled temperament, and antisocial behaviors [7]), and cognitive distortions in luck and control [8,9].

Not surprisingly, gambling problems are highly associated with impaired emotional processes [10]. Among the different personality traits that have been suggested as underlying emotional dysregulation, the construct of alexithymia has gained a wide popularity in the last decades. Affect dysregulation is conceived as the inability to tolerate negative affect by balancing it with positive affect without mostly or solely relying on external objects or behavioral actions [11].

Alexithymia is a multifaceted personality construct that represents a deficit in the cognitive processing of emotion. It is conceived as composed by two higher order factors: deficit of affect awareness (composed of difficulty identifying and describing feelings) and operatory thinking (composed of externally oriented thinking and poor imaginal processes) [12] and has been repeatedly shown to be related to a variety of medical and psychiatric syndromes included in the broader spectrum of disorders of affect regulation. It is strongly influenced by early interactions with caregivers because inadequate responses to the child’s emotions have a major influence on the ability to self-regulate both emotional (through internal working models, ego defenses, self-esteem) and neurobiological (through the autonomic, endocrine, and immune activity) states later in adulthood [12]. Alexithymic individuals show a difficulty in being aware of and expressing their own feelings and in representing experience, behaviors, and mental states in themselves and others. From this theoretical perspective, alexithymia is similar to other psychological constructs that highlight deficits in the functioning of referential activity [13], reflective function [14], and emotional intelligence [15]. The notion that alexithymia can be seen as a personality construct of affect dysregulation is based on several lines of research. For example, neurobiological studies suggested that alexithymia is related to impaired coordination and integration of interhemispheric transfer communication, dysfunction of the right hemisphere, or dysregulation over prefrontal cortex and anterior regions (e.g., anterior cingulate cortex) [16]. Also, a high prevalence rate of alexithymia has been found in a variety of medical and psychiatric disorders of affect regulation such as eating disorders, substance abuse disorders, somatoform disorders, and panic disorder [17]. Evidence so far has shown that the alexithymic deficit in processing feelings is likely to affect mental and somatic health through behavioral actions as ways to regulate affective states (e.g., substance abuse, eating behaviors) or psychophysiological discharge of emotional hyperarousal (e.g., somatization and panic disorder) [18].

To our knowledge, empirical studies on alexithymia and gambling have not been reviewed so far. The aim of this article was therefore to provide a systematic review of empirical studies of the association between alexithymia and gambling.

## 2. Materials and Methods

### 2.1. Study Design and Search Strategy

The Preferred Reporting Items for Systematic Reviews and Meta-Analyses (PRISMA) standards for systematic reviews and meta-analysis were employed for the conduct of the literature search following a systematic and structured approach [19]. Major medical, health and psychological literature databases including PubMed, Cochrane Database for Systematic Review, PsycINFO, Web of Science, and Scopus were used, and the search included all publication years (till April 2019). The keywords used for the systematic search were gambl* AND alexithym*.

### 2.2. Selection Criteria

The inclusion criteria of the studies in this review were original research articles conducted in any population involving any age group which has explored the relationship between gambling problems and alexithymia. Exclusion criteria included reviews, opinion, commentary, and editorials, although their reference lists were searched in turn for any studies not retrieved by the electronic search. Studies which involved gambling tasks not applied to gamblers populations, did not assess directly alexithymia, and published in languages different from English, French, Italian, and Spanish were also excluded. 

### 2.3. Selection Procedure, Data Extraction and Data Management

Upon completion of the search on the electronic databases, titles and abstracts of the identified articles were assessed for their suitability to be included in the review. Additional manual search on Google Scholar was also conducted searching for any further research article not retrieved from the major databases. After assessing the titles and abstracts, the full text of the articles considered suitable were retrieved for further examination of the contents of the studies to determine their final inclusion in the review.

Furthermore, the reference lists of the selected articles were also examined for additional suitable publications that might have been overlooked in the previous search. 

**Figure 1:**
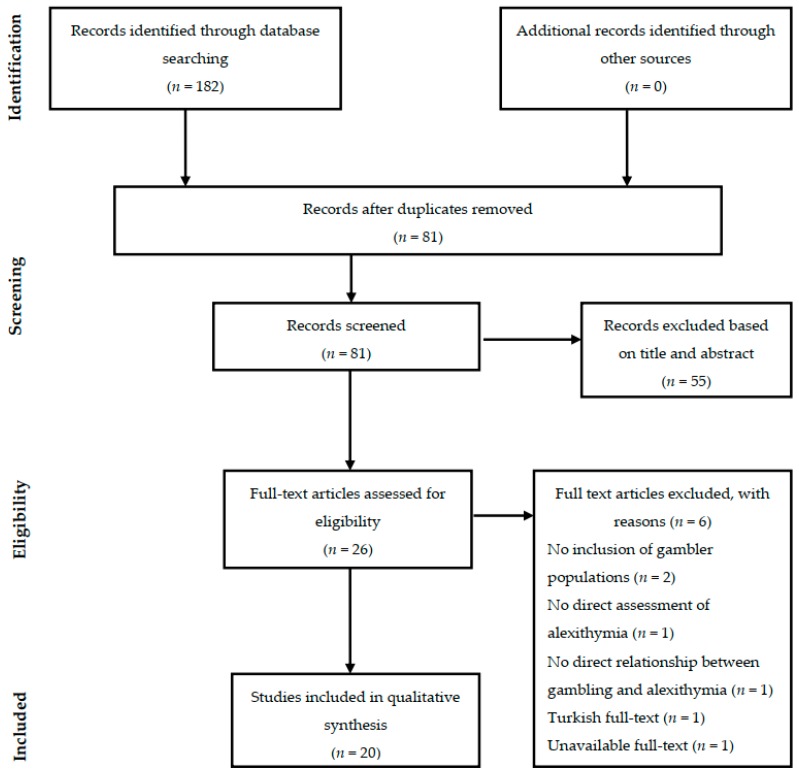
Flowchart of the systematic search.

The search on PubMed, Cochrane, PsycINFO, Web of Science, and Scopus databases initially produced at first 182 articles, 26 of which were selected for a full-text screening and 6 were excluded. No further relevant articles were found during the manual search. Full details of the search process with reasons of exclusion are shown in the PRISMA diagram in Figure 1.

Because of marked differences between the included studies in sample composition, means of assessment of gambling problems, methods for classify participants as alexithymic or non-alexithymic, and statistical analyses, we provide a qualitative synthesis rather than a meta-analysis. Data extraction was carried out under the following headings: sample characteristics, including age, gender and sample size; study design; instruments used; and study findings. Two authors (DM & MCV) independently carried out data extraction. Results were compared, and any discrepancies were resolved by mutual consensus.

## 3. Results

Twenty studies were identified as eligible and retrieved for the present review. Most of the studies (*N* = 15) have been conducted in Europe. Three studies have been conducted in Canada, one in the United States, and one in Australia. All the studies conducted were cross-sectional. Among the included studies only two [20,21] used mediation analyses that allow testing potential paths of association including other variables of interest. There were also no intervention studies targeting gambling problems and alexithymia.

Studies were categorized based on the sample recruited for the study. First, existing research findings on the association between gambling problems and alexithymia in community samples of participants ranging from adolescence to adulthood are summarized below. In a subsequent paragraph, studies on clinical samples of pathological or disordered gamblers recruited in different settings are considered. Fourteen studies were conducted with community samples and six with clinical samples. Table 1 and Table 2 contains a detailed description of all included articles with displayed information about study design, sample, means for the assessment of gambling and alexithymia, other relevant variables evaluated in the relationship between gambling problems and alexithymia, and main relevant results.

In all included studies, alexithymia was assessed with one of the versions of the Toronto Alexithymia Scale, most of which with the 20-item version (TAS-20) [22]. The TAS-20 is the most used assessment instrument for alexithymia and is considered the gold standard in the field. It has a total score and 3 factor scores corresponding to the three facets of alexithymia (DIF, difficulty identifying feelings; DDF, difficulty describing feelings; EOT, externally-oriented thinking), as well as a cutoff score for identifying levels of moderate (50–60) and high (>60) alexithymia. 

Gambling problems were assessed mostly with South Oaks Gambling Screen [20,23,24,25,26,27,28,29,30,31,32,33,34,35,36,37], which is the most used instrument in research literature. In four studies the Canadian Problem Gambling Index [21,38], the Problem Gambling Severity Index [39] or the Kurzfragebogen zum Glücksspielverhalten [29] were used, some of which by adding DSM criteria for gambling disorder.

### 3.1. Studies in Community Samples

Most studies found a higher level of alexithymia in pathological gamblers recruited in community samples (students, community, poker, slot machine, casino, sportsbook, and betting gamblers) compared to problem gamblers or healthy subjects [25,27,32,36,37,40]. When the TAS-20 cutoff scores were used, high alexithymia were found in pathological gamblers in a range of 31–52%. Only one study did not find association between alexithymia and gambling [35]. Moreover, distress and impulsivity mediated the positive relationship between alexithymia and gambling severity [21], while, Di Nicola and colleagues [28] failed to confirm this association in a large sample of adolescents when controlling for impulsivity, anhedonia, and dissociation.

Bibby and Ross [39] provided evidence for understanding a specific aspect of problem gambling, the loss chasing behavior, namely the tendency to continue to bet in an attempt to recover an earlier loss. They found that bettors at risk of problem gambling and high in alexithymia were most likely to chase losses. This means that loss chasing behavior in gamblers may reflect an underlying inability to effectively process emotions. 

Aïte et al. [23] in their cross-sectional study on sportsbook gamblers found a suboptimal performance on the Iowa Gambling Task in alexithymic participants. This means that alexithymia might account for decision making difficulties usually reported in disordered gamblers.

Conflicting results were found related to the specific facets of alexithymia. Some studies found that pathological gamblers have poorer capacity to verbally describe their feelings compared to controls [27,36,40] whilst other studies fail to find significant differences [32,38]. Further, most studies evidenced some degree of impairment in the identification of emotional states in pathological or problem gamblers whereas some studies found within normal-range abilities [27,32,36,38]. Among high-school students, DIF was related to gambling severity through its effect on inability to stop gambling and interpretative bias for both sexes [20]. Similarly, significant differences in EOT were found between pathological gamblers and controls only in few studies [27,32,36]. In relation to loss chasing behavior, DIF was related to both between- and within-session loss chasing, while EOT and DDF were exclusively associated with one type of loss chasing behavior, between-session or within-session, respectively [39].

Some data suggests that alexithymia may be differently involved depending on types of preferred gambling. Although Toneatto and colleagues [40] did not find significant difference between subgroup of gamblers playing different game types, a more recent study [26] found in a racetracks group, but not in casino groups (strategic and non-strategic gamblers), higher levels of alexithymia among pathological gamblers than controls, also after adjusting for depression. Bonnaire and colleagues [27] found that alexithymics had a risk of 4 times higher of belonging to the subgroup of non-strategic pathological gamblers (subjects who play chance or passive games as slot machines) and of 7 times higher to the subgroup of strategic pathological gamblers (subjects whose games involve some element of strategy such as card games) compared to non-alexithymic pathological gamblers.

### 3.2. Studies in Clinical Samples

All but one study were conducted in specialized private and public care centers for addiction behavior. One study was conducted in a naturalistic setting and compared alexithymia levels between pathological gamblers recruited from different gambling venues [24]. Half of the studies compared pathological gamblers with healthy controls [30,33,34]. 

In the two between-group studies, TAS-20 scores indicated significantly higher levels of alexithymia in gambling disorder individuals compared to controls [33,34]. By using the TAS-20 cutoff scores, the prevalence of alexithymia in clinical samples of disordered gamblers ranged from 34% to 67% [24,29,31] depending on studies and gambling types.

Four studies measured the unique effect of alexithymia over and above the variance explained by other relevant variables such as anger expression, attachment, impulsivity, personality disorders and clinical syndromes [30,33,34]. TAS-20 total score was found as a significant predictor of severity of gambling disorder. One study [29] failed to find such association after adjusting for attachment styles with anxiety attachment resulting as the only predictor of gambling severity.

## 4. Discussion

To our knowledge, this is the first review that summarizes the findings on the association between alexithymia and gambling in both community and clinical samples. Our main findings suggest that alexithymia is prevalent in subjects with gambling-related problems in a dose-response relationship, with prevalence of 31–52% in pathological gamblers from community samples and 34–67% in clinical subjects with gambling disorder. According to this finding prevalence rate seems to be higher among clinical disordered gamblers. However, no further consideration on this issue can be done, since no studies have compared pathological gamblers from community and clinical populations and diverse methodologies (e.g., recruitment and diagnostic procedures, use of both categorical and continuous variables, statistical analyses) were used to assess the association between alexithymia and gambling problems. As a further result of the present review, alexithymia may increase symptom severity and the risk for pathological gambling. Finally, alexithymia showed clinically significant interactions with maladaptive personality (sensation-seeking, impulsivity, and aggressiveness), psychopathological (depression, anxiety, and traits of personality disorder), and cognitive (gambling-related cognitions, motivation, strategic, and non-strategic games) factors.

Results are in line with the high alexithymia levels found both in substance use disorders [41,42] and behavioral addiction such as Internet addiction disorder [43] and compulsive buying [44]. It is well-known that high alexithymia levels are associated with higher craving in substance use disorder. Alexithymia scores predict severe tobacco craving during nicotine withdrawal [45], and alexithymic subjects report significantly higher levels of drinking urges compared to the non-alexithymic group [46]. Furthermore, alexithymia-related deficits in emotion identification appear to be positively associated with craving levels reported in response to methamphetamine cues [47]. Findings from our review that alexithymia is prevalent among pathological gamblers suggests that it interacts significantly with other factors in explaining addiction behavior and is not a secondary characteristic of toxic effects of substances, as also indicated by Morie and Ridout [48].

It is largely acknowledged that alexithymia can be seen as a cognitive deficit in the ability of self-regulation of feelings, namely balancing positive and negative affects without the need of an external object or compulsive behaviors. Taylor et al. [11] conceive therefore alexithymia as a trans-diagnostic personality dimension underlying the overarching category of disorders of affect regulation. In particular, it was suggested that addictive behaviors may arise as an attempt of alexithymic subjects to self-regulate their emotions. Overall, the presence of high alexithymia levels could be viewed as a risk factor for control loss in gambling behavior [40], while being aware of one’s own feelings may help to obtain higher impulse control in relation to gambling activities. The role of alexithymia in impairing effective affect regulation can be found also in subjects with sensation-seeking behavior for activities not related to addiction. For example, it has been found in high-risk sport environments as skydiving as an underlying coping strategy for regulating subjective levels of anxiety [49].

Further hypotheses could be provided to explain data on different levels of alexithymia between subgroup of gamblers based on their preferred gambling activity. It is widely recognized that gambling is not an all-or-not phenomenon but can be shaped in different modalities according to the heterogeneity of factors involved in the development of the problematic behavior. For example, Lesieur [50] differentiated between action seekers, having high levels of sensation-seeking and gambling in order to thrill and experience adrenaline and escape seekers gamblers, with high levels of depressed mood and gambling to escape from negative emotional states. In addition, Blaszczynski and Nower [51] differentiated between three subtypes of pathological gamblers, namely the behaviorally conditioned, the emotionally vulnerable, and the antisocial impulsivist. In particular, the second pathway characterizes subjects who used gambling in order to escape from dysregulated mood states while the third subtype characterizes subjects with high impulsivity, low tolerance for boredom, and antisocial personality traits who used gambling in order to increase emotional arousal. Of interest, our review showed that strategic gamblers who prefer games involving active playing strategies (i.e., poker) may be inclined to play in order to increase emotional arousal and bodily sensations [26]. Conversely, the underlying motivation for choosing of non-strategic games in alexithymic individuals would be driven by the need to use passive strategies (i.e., chance, slot machines) to cope with negative emotions experienced as a consequences of gambling losses [27,39,52].

A number of limitations limit the generalizability of findings on the association of alexithymia with problematic gambling behavior. Studies published in the last two decades have used a cross-sectional design that makes it difficult to determine the direction of causality. Alexithymia may be a causative factor for developing gambling behavior or the consequence of maladaptive habits of wagering. A third independent factor as comorbid psychopathology or social environment may also explain the association of alexithymia and problematic gambling. Longitudinal studies are therefore needed to ascertain whether alexithymia is an overtime stable primary feature that contributes to the maintenance of the addiction behavior. Also, all reviewed studies utilized a convenience/purposive sampling procedure. This limits the possibility to generalize the results to more carefully selected samples from different clinical and specific settings. For example, the dose-response increase of alexithymia from gamblers in community to clinical subjects with diagnosed gambling disorder suggests the use of different assessment strategies for identifying problematic gambling. Future research should explore the role of possible not yet investigated mediator and/or moderator factors that may account for this association as the environmental background of gamblers or the shared and non-shared genetic factors. 

Potential limitations also include the difficulty to have access to non-published data, which could have biased this review. Although a manual search in Google Scholar was performed, we can assume that data that were not published at all or published only in the grey literature not indexed in Google Scholar may be missed in this review, raising the issue of publication bias. This possible limitation of the present review should be kept in mind when generalizing the results to the gambling population as a whole. To further measure the strength of the association between alexithymia and gambling problems and to integrate published and non-published results, randomized controlled trials and meta-analysis are suggested. 

In conclusion, results identified through this review suggest, that alexithymia should be considered in the clinical assessment of gambling behavior. Since alexithymia has been found to respond positively to psychological interventions [53] and gambling problems can also be effectively reduced with multiple strategies of care [4], assessing alexithymia in these subjects may help in planning more effective individually tailored treatment protocols. Gamblers may benefit from focusing on recognizing and making sense of their own emotional states and to develop more adaptive ways to manage their feelings [54].

## Figures and Tables

**Table 1:** Studies on alexithymia and gambling in community samples.

Authors	Country	Study Design	Sample	Assessment of Gambling	Assessment of Alexithymia	Other Relevant Variables	Main Results
Lumley and Roby (1995) [32]	USA	Cross-sectional	- University students - N = 1147 - M = 456 - F = 691 - Age (median) = 19	- SOGS	TAS-26	- Depression - Physical illness	- Higher prevalence of higher-level alexithymia in PG (31.4%) than HC participants (11.1%), (χ^2^ = 13.2, *p* <0.001). - Between-group comparisons showed that PG subjects scored significantly higher than HC on the TAS total (even after controlling for physical illness) and EOT and PrG on DIF than HC, controlled for depression, and physical illness.
Parker et al. (2005) [36]	Canada	Cross-sectional	- University students - N = 562 - M = 113 - F = 449 - Age = 19.86 ± 3.04	- SOGS	TAS-20	- Mood	- Between-group comparisons showed that PG subjects scored significantly higher on the TAS total as well as DDF and EOT than HC, either unadjusted or after controlling for mood scores.
Toplak et al. (2007) [37]	Canada	Cross-sectional	- Community sample - N = 107 - M = 107 - F = 0 - Age = 31.8 ± 11.8	- DSM-IV criteria-based questionnaire - SOGS	TAS-20	- Cognitive ability	- PG subjects scored higher to the TAS than HC (F(2, 104) = 11.03, *p* < 0.001), even after controlling for age and cognitive ability (F(2, 104) = 7.61, *p* < 0.001). TAS-20 accounted for the most unique variance of gambling behavior (*R*^2^ Change = 0.104, F = 15.53, *p* < 0.001) in hierarchical regression, after age and cognitive ability were partialled out.
Toneatto et al. (2009) [40]	Canada	Cross-sectional	- Community sample - N = 296 - M = 130 - F = 166 - Age = 43.4 ± 11.9	- DSM-IV criteria	TAS-20	- Gambling types - Impulsivity	- TAS total, DIF, and DDF scores significantly higher in PG, PrG, and HC (F(2, 293) = 16.4, *p* < 0.001; F(2, 293) = 20.7, *p* < 0.001; F(2, 293) = 16.8, *p* < 0.001 respectively). - With impulsivity specified as covariate, the three groups still differ only on DDF score (F(2, 293) = 6., *p* < 0.01). - Alexithymia scores tended to increase significantly with intensification of gambling problems (*p* < 0.05). - Male PG scored higher than females on DDF (t(141) = 2.2, *p* < 0.05) and EOT (t(141) = 3.1, *p* < 0.01). - Total number of DSM symptoms significantly higher among high alexithymics for PG, but not for PrG. - No significant difference in TAS score when comparing sub-groups of gambling types.
Mitrovic and Brown (2009) [38]	Australia	Cross-sectional	- Community sample (poker gamblers) - N = 96 - M = 75 - F = 18 - Age = 27.3 ± 8.25	- CPGI	TAS-20	- Gambling cognitive distortions - Motivation toward gambling - Skill- and non-skill gambling	- Positive association of CPGI with TAS-20 total (*r* = 0.26), DIF (*r* = 0.39), and DDF (*r* = 0.22) scores, but not with EOT (*r* = 0.10). - The only alexithymic dimension that discriminated between PrG and HC was DIF (F(2, 96) = 8.1, *p* < 0.05). - When considered as possible predictor of gambling problems among poker players with gambling cognitive distortions, motivation toward gambling, skill- and non-skill gambling, alexithymia did not significantly contribute to the model.
Bonnaire et al. (2010) [25]	France	Cross-sectional	- Community sample (slot machine gamblers) - N = 64 - M = 28 - F = 36 - Age = 34.6 ± 6.3	- DSM-IV criteria - SOGS	TAS-20	- Depression - Gambling types - Sensation seeking	- The prevalence of alexithymia was significantly higher among participants classified as PG (44%) than RG (28%) and NRG (5%) (χ^2^ = 13.2, *p* < 0.001).
Bonnaire et al. (2013) [26]	France	Cross-sectional	- Community sample (gamblers recruited from racetracks and casino) - N = 186 - M = 150 - F = 36 - Age = NR	- DSM-IV criteria-based questionnaire - SOGS	TAS-20	- Depression - Gambling types	- Within the racetracks and slot machines subgroups, PG scored higher than HC on TAS-20 total (racetracks: F(1, 78) = 10.47, *p* < 0.01; slot machines: F(1, 63) = 4.78, *p* < 0.05) and DIF (racetracks: F(1, 78) = 10.91, *p* < 0.01; slot machines: F(1, 63) = 4.39, *p* < 0.05) scales. - Between-group comparison within the traditional casino game subgroup did not reveal any differences in TAS-20. - Adjusting for depression in ANCOVA, TAS-20 scores were not different in slot machines gamblers whereas racetracks gamblers continued to score higher (TAS-20 total: F(1, 78) = 6.00, *p* < 0.05; DIF: F(1, 78) = 5.83, *p* < 0.05). - Categorical analysis showed that the prevalence of alexithymia (TAS-20 > 60) was significantly higher among racetracks PG than slot machines and traditional games PG (67% vs. 44% and 34%, *p* = 0.04, respectively). - Significant correlations were found between DIF and depression scores in the slot machines PG group (*r* = 0.54) and between TAS total and depression scores in the racetracks PG group (*r* = 0.35).
Cosenza et al. (2014) [20]	Italy	Cross-sectional	- High-school students - N = 546 - M = 273 - F = 273 - Age = 18.1 ± 0.5	- SOGS-RA	TAS-20	- Gambling-related cognitions	- Weak significant association between gambling severity and TAS total (*r* = 0.14), DIF (*r* = 0.18), and DDF (*r* = 0.11). - DDF was significantly associated with gambling severity (*R*^2^ Change = 0.044, *p* = 0.03) after accounting for gender and gambling-related cognitions (i.e., inability to stop gambling and interpretative bias). - Mediation analysis revealed that DIF contributed to gambling severity through its influence on inability to stop gambling (*z* = 4.97, *p* <0.001) and interpretative bias (*z* = 3.52, *p* <0.001). Both indirect effects were significant for males and females.
Aïte et al. (2014) [23]	France	Cross-sectional	- Community sample (sportsbooks gamblers) - N = 28 - M = 25 - F = 3 - Age = 34.6 ± 6.3	- DSM-IV criteria - SOGS	TAS-20	- Decision making - Anxiety - Depression	- TAS total score correlated significantly with decision making performance in PG (*r* = −0.45) even when controlling for the effects of anxiety and depression, (*r* = −0.44). - Comparing alexithymic PG, non-alexithymic PG, and HC a significant lower decision making was found for alexithymic PG compared with the other two groups (*d* = −1.28 and *d* = −1.47, respectively).
Montel et al. (2014) [35]	France	Cross-sectional	- Community sample N = 77 - M = 43 - F = 34 - Age = NR	- SOGS	TAS-20		- No significant differences were found on alexithymia scores between online PG, PrG, and HC.
Bibby and Ross (2017) [39]	United Kingdom	Cross-sectional	- Community sample (betting gamblers) - N = 58 - M = 50 - F = 8 - Age = 48.1 ± 13.46	- PGSI	TAS-20	- Loss-chasing behavior	- A significant positive correlation was found between TAS total and gambling problems scores (*r* = 0.46). - Participants at risk of problem gambling were twice as likely to be at or near caseness for alexithymia as those at low risk for problem gambling. - Alexithymia accounted for approximately 21% of the variance in gambling problems. - Participants at or near caseness for alexithymia showed a statistically difference between the proportion of bets after a loss and after a win (5.6%). This difference was not found for non-alexithymic gamblers. - Between- and within-session loss chasing were associated with TAS-20 total score. DIF and EOT scores were related to between-session loss chasing, while DIF and DDF were related to within-session loss chasing.
Di Nicola et al. (2017) [28]	Italy	Cross-sectional	- High-school students - N = 996 - M = 240 - F = 756 - Age = 18.1 ± 0.5	- SOGS-RA	TAS-20	- Anhedonia - Dissociation - Impulsivity	- No association was found between gambling problems and alexithymia when impulsivity, anhedonia, and dissociation variables were controlled for.
Bonnaire et al. (2017) [27]	France	Cross-sectional	- Community sample (gamblers recruited from different gambling venues) - N = 226 - M = 190 - F = 36 - Age = 32.6 ± 8.1	- DSM-IV-TR criteria - SOGS	TAS-20	- Depression - Strategic and non-strategic gambling	- Compared with HC, PG had higher TAS total score (controlled for depression: F = 20.18, *p* < 0.001; controlled for gender: F = 13.00, *p* < 0.001), DIF score (controlled for depression: F = 28.17, *p* < 0.001; controlled for gender: F = 11.26, *p* < 0.001), DDF score (controlled for depression: F = 9.60, *p* < 0.001; controlled for gender: F = 6.98, *p* < 0.001), and EOT score (controlled for depression: F = 3.83, *p* < 0.05, controlled for gender: F = 6.22, *p* < 0.01). - Significantly more PG than HC scored in the high-level range of TAS-20 (≥56) (51.9% vs. 20.0%, χ^2^ = 25.17, *p* < 0.001). - Alexithymic PG had a higher severity of gambling problems (SOGS: F = 21.94, *p* < 0.001; DSM-IV criteria: F = 21.43, *p* < 0.001) than non-alexythimic PG. - Logistic regression found that being alexithymic was highly associated with PG together with age and being depressed (OR = 4.206, 95% CI = 2.261–7.825, *p* < 0.001) and that DIF was more weakly associated with PG together with age (OR = 1.067, 95% CI = 1.004–1.135, *p* < 0.05). - In strategic gamblers, being alexithymic (OR = 6.804, CI = 2.534–18.261, *p* < 0.001) and DIF (OR = 1.121, CI = 1.014–1.240, *p* < 0.05) were positively associated with PG. - In non-strategic gamblers alexithymia was not associated with PG when evaluated through a multiple logistic regression.
Noël et al. (2018) [21]	Belgium	Cross-sectional	- Community sample (gamblers) - N = 106 - M = 106 - F = 0 - Age = 31.55 ± 10.36	- CPGI	TAS-20	- Distress - Impulsivity - Working memory	- Alexithymia was a significant predictor of gambling severity and its effect was fully mediated by distress and impulsivity (indirect effect: β = 0.29; 95% bootstrap CI = 0.13, 0.44).

CPGI: Canadian Problem Gambling Index; DDF: Difficulty Describing Feelings; DIF: Difficulty Identifying Feelings; EOT: Externally Oriented Thinking; F: Females; HC: Healthy Controls; M: Males; NRG; Non-Regular Gamblers; NR: Not Reported; PG: Pathological Gamblers; PrG: Problem Gamblers; PGSI: Problem Gambling Severity Index; RG: Regular Gamblers; SOGS: South Oaks Gambling Screen; SOGS-RA: South Oaks Gambling Screen Revised for Adolescents; TAS: Toronto Alexithymia Scale.

**Table 2:** Studies on alexithymia and gambling in clinical samples.

Authors	Country	Study Design	Sample	Assessment of Gambling	Assessment of Alexithymia	Other Relevant Variables	Main Results
Bonnaire et al. (2009) [24]	France	Cross-sectional	- PG recruited from different gambling venues (cafés, racetracks, slot machines, and traditional casino games) - N = 141 - M = 126 - F = 15 - Age = NR	- DSM-IV criteria - SOGS	TAS-20	- Depression - Sensation seeking	- PG racetrackers with high-level alexithymia were more prevalent than PG slot machines and traditional casino games subjects (67% vs. 44% and 34%, respectively) (*p* = 0.04). - Significant positive correlation between DIF and disinhibition scores (*r* = 0.36), and between TAS total and depression scores (*r* = 0.35) in PG racetrackers. - Significant negative correlation between TAS-20 total and sensation seeking total scores (*r* = 0.47) and positive correlation between DIF and depression scores (*r* = 0.35) in PG slot machines gamblers.
Grall-Bronnec et al. (2010) [31]	France	Cross-sectional	- Clinical sample of PG - N = 24 - M = 19 - F = 5 - Age = 43.8 ± 10.7	- DSM-IV criteria - SOGS	TAS-20		- Two third of participants (66.7%) scored in the alexithymia range (TAS-20 > 56).
Maniaci et al. (2015) [33]	Italy	Cross-sectional, case-control study	- Clinical PG and control samples - N = 140 (70 PG and 70 HC) - M = 118 - F = 22 - Age = PG 42.41 ± 10.51; HC 41.28 ± 13.55	- SOGS	TAS-20	- Clinical syndromes - Personality disorders	- Significant higher scores were observed in PG compared to HC on TAS total (F(1, 138) = 13.656, *p* < 0.001), DDF (F(1, 138) = 8.470, *p* < 0.01), and EOT (F(1, 138) = 16.741, *p* < 0.001) scores. - Hierarchical multiple regression showed that alexithymia significantly predicted gambling severity (*R*^2^ Change = 0.052; F(4, 109) = 4.725, *p* < 0.01) over and above the variance explained by personality disorders and clinical syndromes in the first step. DDF was the only significantly TAS-20 predicting scale (β = −1.612, *p* = 0.04).
Gori et al. (2016) [30]	Italy	Cross-sectional, case-control study	- Clinical PG and control samples - N = 204 (154 PG and 50 HC) - M = 178 - F = 26 - Age = 47.75 ± 12.08	- SOGS	TAS-20	- Dissociation - Impulsivity	- Gambling severity was significantly and positively correlated with TAS total score (*r* = 0.50). - TAS-20 total score was a significant predictor (β = 0.241, *p* < 0.001) of gambling severity explaining, together with impulsivity, 40% of its variance. - Only the DIF factor was shown as a significant predictor (β = 0.392, *p* < 0.001) of gambling severity (Adjusted *R*^2^ = 0.13).
Maniaci et al. (2017) [34]	Italy	Cross-sectional, case-control study	- Clinical PG and control samples - N = 200 (100 PG and 100 HC) - M = 170 - F = 30 - Age = PG 41.53 ± 10.96; HC 41.27 ± 13.46	- SOGS	TAS-20	- Anger expression	- PG subjects scored higher than HC to all TAS-20 scales (Total: F = 26.053, *p* < 0.001; DIF: F = 4.808, *p* < 0.05; DDF: F = 17.525, *p* < 0.001; and EOT: F = 28.932, *p* < 0.001). - Positive significant association between TAS-20 and gambling severity was found (*r* = 0.46) and in multiple regression alexithymia significantly predicted gambling severity (β = 0.457, *p* < 0.001), accounting for 20.9% of its variance (F(1, 198) = 52.319, *p* < 0.001).
Di Trani et al. (2017) [29]	Italy	Cross-sectional	- Clinical DG - N = 60 - M = 48 - F = 12 - Age = 44.53 ± 13.00	- KFG - SCID 5 RV	TAS-20	- Attachment style	- High-level (TAS-20 ≥ 61) and borderline-level (TAS-20 = 51–60) of alexithymia was found in 40% and 37%. - No significant relationships were found between alexithymia (total and factor scores) and gambling severity. - TAS total score was not found to be a significant predictor of gambling severity through a multiple regression analysis executed with age, gender, and attachment variables.

DDF: Difficulty Describing Feelings; DG: Disordered Gamblers; DIF: Difficulty Identifying Feelings; EOT: Externally Oriented Thinking; F: Females; HC: Healthy Controls; KFG: Kurzfragebogen zum Glücksspielverhalten; M: Males; NR: Not Reported; PG: Pathological Gamblers; SCID 5 RV: Structured Clinical Interview for the DSM-5 Research Version; SOGS: South Oaks Gambling Screen; TAS: Toronto Alexithymia Scale.
